# Co-Incidence of Acute Appendicitis and Appendiceal Transection after Blunt Abdominal Trauma: A Case Report

**Published:** 2013-12

**Authors:** Sam Moslemi, Hamid Reza Forootan, Maryam Tahamtan

**Affiliations:** 1 Department of General Surgery, School of Medicine, Shiraz University of Medical Sciences, Shiraz, Iran;; 2 Department of Pediatric Surgery, School of Medicine, Shiraz University of Medical Sciences, Shiraz, Iran;; 3 Department of Internal Medicine, School of Medicine, Shiraz University of Medical Sciences, Shiraz, Iran

**Keywords:** Abdominal injury, Appendiceal, Transection, Acute appendicitis

## Abstract

A 13-year-old boy with a history of bicycle handlebar injury to the left side of his abdomen was brought to the Emergency Department of our center. On admission, his vital signs were stable and abdominal examination revealed ecchymosis and tenderness of the injured areas. Mild to moderate free fluid and two small foci of free air in the anterior aspect of the abdomen, in favor of pneumoperitoneum, were detected in abdominopelvic sonography and CT-scan, respectively. In less than 6 hours, the patient developed generalized peritonitis. Therefore, exploratory laparotomy was promptly performed, which revealed appendiceal transection and rupture of the small bowel mesentery. Appendectomy and small bowel mesoplasty were done, with pathological diagnosis of acute appendicitis and periappendicitis. After surgery, the patient had a non-complicated hospital course. This rare case highlights the significance of the early management of appendiceal traumatic injuries in order to prevent further complicated events, especially in patients who are much more exposed to this risk due to their traumatic background.

## Introduction

Trauma and acute appendicitis are the two most common entities of childhood surgical emergencies.^1^ Isolated appendiceal traumatic injuries, especially in the context of a blunt abdominal trauma, are strikingly rare and many cases remain unreported due to concurrence with other intra-abdominal injuries.^2^ Despite the fact that the underlying mechanism of acute appendicitis has yet to be fully elucidated, it is presumed that luminal obstruction by an internal or external compression such as faecalith or lymphoid hyperplasia, respectively, is the key point in pathogenesis. Abdominal trauma is assumed to have an indirect causative role.^3^ There is still doubt as to the direct pathogenic effect of abdominal trauma in acute appendicitis;^4^^-^^6^ nevertheless, further appendiceal injuries would render the situation far more challenging. This report presents a 13-year-old boy with manifestations of generalized peritonitis within a few hours after a blunt abdominal trauma, in whom exploratory laparotomy revealed an appendiceal transection.

## Case Report

A 13-year-old boy was brought to the Emergency Department of Nemazee Hospital, Shiraz, Iran, at 10:00 p.m., June 1^st^, 2012, following a collision between his bicycle and a motorcycle, during which the patient had the left side of his abdomen injured by his bicycle handlebar. On admission, the patient only complained of left flank pain. 

**Figure 1:**
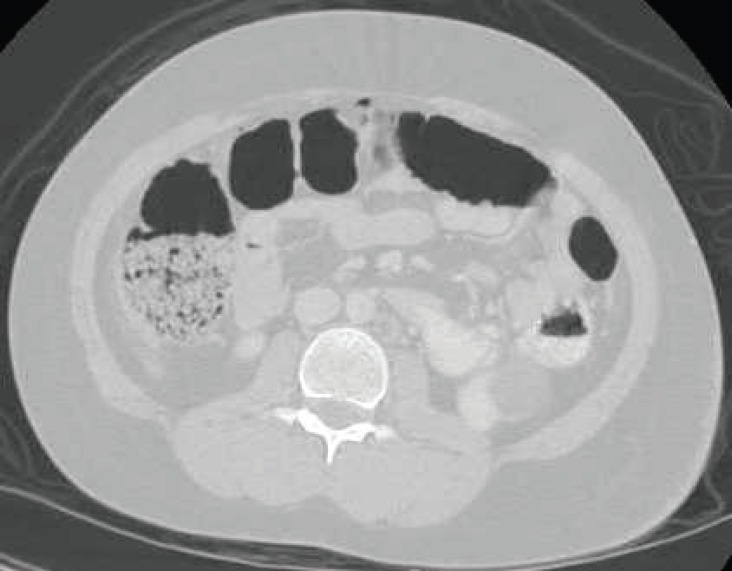
This computed tomographic scan of the abdominopelvic cavity demonstrates small foci of air in the anterior aspect of the abdomen, in favor of traumatic pneumoperitoneum

A review of his surgical history was remarkable for two previous surgical operations; i.e. tonsillectomy and right inguinal herniorrhaphy. He did not mention any abdominal discomfort of recent duration. On arrival, he was thoroughly conscious, was not tachypneic, and had an axillary temperature of 37°C, blood pressure of 110/70 mm Hg, and heart rate of 90 bpm with no orthostatic change. Mild ecchymosis of his periumbilical area and left flank was visible. The bowel sounds were normally audible. Abdominal palpation revealed mild to moderate tenderness in the same ecchymotic areas. A complete blood count (CBC) yielded a hemoglobin count of 13.5 gr/dl, white blood cell count (WBC) of 14700/mm^3^, and platelet count of 313000/mm^3^. Other blood tests and urinalysis were normal. The findings of plain chest and abdominal graphies were inconclusive, and abdominopelvic sonography showed mild to moderate free fluid in the abdominopelvic cavity. Spiral abdominopelvic CT-scan revealed two small foci of air in the anterior aspect of the abdomen, in favor of pneumoperitoneum. Two areas of faint increased density and fat stranding in the subcutaneous fat of the left side of the abdomen and another one in the anterior right side of the abdomen were detected, all due to post-traumatic changes. A small area of increased opacity, resembling hematoma, was seen inferior to the spleen and lateral to the psoas muscle in left lower abdominal quadrant (figure 1). Within less than 6 hours, the patient abruptly developed diffuse abdominal pain, accompanied by an axillary temperature of 38°C, mild tachycardia, hypoactive bowel sounds, and generalized abdominal rigidity and tenderness. Generalized rebound tenderness was also present. No significant hemoglobin drop was noted. Due to high suspicion of peritonitis, exploratory laparotomy was performed via an upper midline incision, which revealed transection of the appendix from its distal half and some foci of the small bowel mesentery’s rupture. About 100 cc fresh blood was sucked, the appendix was dissected from the mesoappendix, and small bowel mesoplasty was performed. No evidence of fibrinopurulent peritonitis or solid organ injury was detected. The pathological examination of the appendix demonstrated acute appendicitis with periappendicitis. The patient experienced an uncomplicated postoperative hospital course and was discharged after 5 days.

## Discussion

The appendix is a highly mobile, small structure so that it is rarely affected by direct blunt abdominal trauma.^4^ It is thought that there is a multifactorial mechanism indirectly contributing to blunt trauma-induced acute appendicitis. As a complication of internal or external blood loss, visceral hypoperfusion occurs. The following reperfusion will lead to visceral edema, resulting in a rise in intra-abdominal pressure (IAP). This condition is deteriorated by extensive fluid resuscitation and diminished size of the abdominal cavity by other traumatic complications such as intraperitoneal bleeding or free air occupying space, retroperitoneal hematoma, acute gastric dilatation, and external compression. An abrupt rise in IAP increases intracecal pressure and a subsequent rapid distension of the appendix, resulting in mucosal abrasion. Both mucosal abrasion and decreased blood flow will end in acute appendicitis and its complications.^7^ Despite this complex mechanism, the association between acute appendicitis and blunt abdominal trauma is still controversial. Due to their high prevalence, some have considered their coexistence as an incidental event, while others have argued that acute appendicitis may cause the patient to be vulnerable to a traumatic event.^4^ In our case, visceral hypoperfusion and resultant increased IAP does not seem to have a pathophysiological role due to the absence of a significant volume loss.

Appendiceal rupture after blunt abdominal trauma is also exceedingly rare. Whether appendiceal rupture occurs as a complication of advanced acute appendicitis or as a consequence of direct blunt abdominal trauma has yet to be fully clarified. In our case, concurrence of appendiceal rupture and acute appendicitis rendered it difficult to determine which one was prior to the other. As we mentioned, the patient had been asymptomatic before the trauma and there was no histopathological evidence of advance acute appendicitis to be responsible for the subsequent appendiceal rupture. Furthermore, consideration of transaction as an antecedent event does not justify the pathologic report of inflammation because of trauma-induced vascular injury and tissue ischemia. 

Appendiceal rupture was first reported in 1938 with a two-week history of pneumatic drill use resting on the right iliac fossa.^8^ In 1977, a 30-year-old man was reported to have developed acute abdominal pain two days after a blunt severe direct trauma to the abdomen. Surgical exploration revealed appendix avulsion from its distal three quarters with fibrinopurulent mucosa and surrounding bruising of the cecal wall. Consequently, appendectomy and caecostomy were performed. Nonetheless, the patient experienced a complicated postoperative course due to the formation of multiple subcutaneous parastomal abscesses and resultant septicemia.^2^ Reviewing the literature lists a few other such conditions.^9^^-^^11^ However, we found only one case of bicycle handlebar injury presented by acute appendicitis. In the said case, the bicycle handlebar had injured the lower abdomen and symptoms started 2 days after the trauma with the diagnosis of perforated suppurative appendicitis in pathological examination.^12^ The appendiceal transection in our case is in fact a contrecoup injury due to the opposite primary side of the handlebar harmful contact, which was visible in the left part of the patient’s abdomen. It is also worthy of note that in the majority of the available reports, late presentation of symptoms features prominently. Furthermore, in a patient with trauma, diagnosis of acute appendicitis is difficult and may cause delay in early management.^13^ It may contribute to more complex pathologic forms of acute appendicitis. In our case, rapid development of the symptoms and signs of generalized peritonitis hinted at chemical peritonitis, which was subsequently confirmed by our observations during exploratory laparotomy. Our early management precluded such further problems as fibrinopurulent peritonitis and its complications.

## Conclusion

Despite the hitherto uncertain relationship between blunt abdominal trauma and acute appendicitis, consideration of this rare co-existence would result in early management and an uneventful postoperative course. 
